# Convalescent Hospital Use among Young and Older Female Cancer Survivors

**DOI:** 10.3390/ijerph18052744

**Published:** 2021-03-08

**Authors:** Hyesun Park, Kisook Kim

**Affiliations:** 1 Department of Nursing, Baekseok Culture University, Baekseokdaehakro 1, Dongnam gu, Cheonan 31065, Korea; april1007@naver.com; 2 Department of Nursing, Chung-Ang University, Heukseokro 84, Dongjak gu, Seoul 06974, Korea

**Keywords:** women cancer survivors, convalescent hospital, utilization, medical expense

## Abstract

This study analyzed national health data to assess convalescent hospital use among female cancer survivors according to age group. This retrospective study collected data from women recovering from breast, colon, and stomach cancer, based on health insurance claim data over 5 years (2013–2017), from the Korea Health Insurance Review and Assessment Service (KHIRA). Interestingly, the number of young and older women who were treated in convalescent hospitals increased every year. In addition, total medical expenses increased in both groups. The annual rate of convalescent hospitalization was higher in older women (8.29~16.39%) than in younger women (4.01~7.46%). The total yearly medical expenses of cancer survivors in convalescent hospitals increased in both age groups and all cancer types, and the range of increase rate was from 7.7% in young breast cancer survivors to 32.2% in young colon cancer survivors. Visit days and days of medication increased noticeably in young colon cancer survivors. Taken together, these data have confirmed the importance of developing standard guidelines for inpatient management in convalescent hospitals and the health management of women cancer survivors by cancer type. This includes establishing a health management system and medical policies.

## 1. Introduction

Cancer is a major public health concern worldwide; if Korean women reach their average life expectancy (86 years old), one in three will have developed cancer [1]. The incidence rate of cancer is higher in women than men until their early 50s. Further, approximately 1 in 11 women aged 65 years have experienced cancer, and the 5-year survival rate is increased in women (77.5%) when compared with men (63.5%) [1]. This suggests that techniques are required to maintain health while living as cancer survivors for a long time [2].

Cancer patients experience body function depression, such as bone marrow suppression, nausea, vomiting, diarrhea, and neuropathy due to immune system damage [3,4]. Additionally, short- and long-term complications of the endocrine system, such as hormonal dysfunction, are present after cancer treatment [5]. Cancer survivors develop unresolved symptoms, such as pain, fatigue, anxiety, and depression, after diagnosis and new health problems, such as cardiovascular disease and endocrine disorders [5]. They must live with the long-term complications of cancer and treatment; therefore, regularly checking and managing symptoms is a very important task [6]. The long-term symptoms of cancer, combined with the ongoing management of other chronic conditions, make survivors particularly vulnerable to taking multiple medications and their side effects, including drugs with abuse potential and medication expenditures [7,8].

Young female cancer patients generally experience early menopause during treatment and have high demands for information on childbirth and future birth-related factors and disease management [7]. Moreover, fear and anxiety about recurrence increases with age; therefore, psychosocial attention and management of young survivors is required [2,8]. Healthy lifestyle habits, such as maintaining a normal weight, eating a healthy diet, and physical activity, improves the prognosis and quality of health-related life of cancer survivors, particularly for older patients [9]. Conversely, poor eating habits can be life threatening [10]. Furthermore, emotional support and healthy diet intake affects physical and psychological well-being [11]. Taken together, these reports show a difference in health care needs according to the age and lifestyle of female cancer survivors.

Long-term hospitalization treatment is difficult, except for surgery or severe conditions at tertiary and acute medical institutions; therefore, many cancer survivors in Korea use convalescent hospitals for psychological support, counseling, and convalescence [12]. Despite these changes, most research on female cancer survivors is focused on patients in tertiary medical institutions, limited to specific periods, such as the initial diagnosis or the stage of chemotherapy, and is focused on breast cancer [13,14].

Convalescent hospitals provide programs tailored to the needs of cancer patients and integrate complementary and alternative medicine and immunotherapy; therefore, they are used after cancer diagnosis and during chemotherapy and radiation therapy treatments or after treatment is complete [12,15]. These hospitals are recognized as a place for long-term care and treatment for the elderly [16]; however, the correlation between cancer incidence and age [17] has led to a need for a better understanding of convalescent hospital use by cancer survivors. 

The increased incidence in cancer in women and the corresponding increase in morbidity can lead to adverse effects, such as an increase in national medical expenses and a decrease in quality of life for women. Therefore, the management of female cancer survivors is an important national health management project. This study investigated convalescent hospital use among women recovering from breast, colon, and stomach cancer by age group. These are the three cancer types with the highest incidence in women in Korea, based on national health data. 

## 2. Materials and Methods

This study has a retrospective design, which investigated convalescent hospital use in women who had survived breast, colon, or stomach cancer. We used health insurance claim data for 5 years (2013–2017) from the Korea Health Insurance Review and Assessment Service (KHIRA).

Female patients who visited convalescent hospitals during 1 January 2013 to 31 December 2017, and had the disease codes for breast cancer (C50), colon cancer (C18–21), or were treated with gastric cancer (C16) as their main disease (from the Korean standard disease classification [18]), were included in this study. Women aged ≤50 and >50 years were classified as the young and older groups, respectively. This age cut-off was used based on previous studies [19] that use this as a criterion for distinguishing fertility, physical, and psychosocial functions.

Data was requested from KHIRA regarding the current status of medical use in convalescent hospitals for female patients who had been treated for breast, colon, or stomach cancer between 2013 and 2017 and who had claimed health insurance. These data were exempt from deliberation by the Institutional Review Board at C University because they did not contain any personal information. Data were collected regarding those patients who received treatment with the appropriate disease code on an annual basis, excluding oriental medicine, psychiatric, and medical rehabilitation hospitals. Hospital use and total medical expenses were collected as the total medical care benefits billed annually. These expenses included statutory patient co-payments and excluded non-payments. The days of visit referred to the number of days that patients visited or remained in convalescent hospitals. The days of medication referred to the number of days of medication during visits or hospitalization. 

Data were analyzed using descriptive statistics, such as frequency and percentage, average, and annual average rate of increase. Total medical expenses, patient numbers, visit days, and days of medication were calculated per year, and the total medical expenses per person receiving treatment (Korea won (KRW)), visit days (days), and days of medication (days) were calculated per patient numbers.

## 3. Results

### 3.1. Hospitalization of Female Cancer Survivors by Age Group

Table 1 shows the number of female cancer survivors in convalescent hospitals by age group. The average annual rate of increase in the young group was 8.59%, 7.46%, and 4.01% in colon, gastric, and breast cancer, respectively. The average annual rate of increase in the older group was 16.39%, 11.49%, and 8.29% in breast, stomach, and colon cancer, respectively.

### 3.2. Total Medical Expenses for Female Cancer Survivors in Long-Term Care Hospitals by Age Group

Table 2 shows the total yearly medical expenses of cancer survivors in convalescent hospitals by age group. These increased in both age groups and all cancer types. In young breast cancer survivors, there was an increase of 7.7%, from 4,972,000 KRW in 2013 to 5,356,000 KRW in 2017. In older breast cancer survivors, there was an increase of 12.3%, from 5,542,000 KRW in 2013 to 6,224,000 KRW in 2017. In young women colon cancer survivors, there was an increase of 32.2%, from 3,912,000 KRW in 2013 to 5,171,000 KRW in 2017. In the older group, there was an increase of 20%, from 6,027,000 KRW to 7,231,000 KRW. In young women gastric cancer survivors, there was an increase of 19.9%, from 3,794,000 KRW in 2013 to 4,355,000 KRW in 2017. In the older group, there was an increase of 16.6%, from 5,237,000 KRW to 6,107,000 KRW.

### 3.3. Visit Days for Female Cancer Survivors in Long-Term Care Hospitals by Age Group

Table 3 shows the number of days of visit of women cancer survivors to the convalescent hospital according to the age group by year. The total number of visit days of young patients who were treated for breast cancer increased from 191,610 days in 2013 to 249,870 days in 2017; the number of visits per person remained the same (99 days in 2013 to 98 days in 2017). The total number of visit days for older cancer survivors increased from 273,948 days in 2013 to 538,563 days in 2017; visits per person increased from 100 days in 2013 to 107 days in 2017.

The number of visit days for young patients who received treatment for colon cancer increased from 14,277 days in 2013 to 25,168 days in 2017; visits per person increased from 74 days to 94 days. The number of visit days for older survivors increased from 256,966 days in 2013 to 393,834 days in 2017; visits per person increased from 91 days to 102 days.

The number of visit days for young patients who were treated for gastric cancer increased from 20,334 days in 2013 to 30,317 days in 2017; visits per person increased from 71 to 80 days. The number of visit days in the older group increased from 152,901 days to 232,608 days; visits per person increased from 80 days to 87 days.

### 3.4. Days of Medication for Female Cancer Survivors in Long-Term Care Hospitals by Age Group

Table 4 shows the number of days of medication of female cancer survivors receiving treatment at convalescent hospitals by year by age group. For young survivors diagnosed with breast cancer, this increased from 194,352 days in 2013 to 259,978 days in 2017; per person this increased from 100 days to 102 days. The number of medication days for older survivors increased from 277,576 days to 554,693 days; per person this increased from 101 days to 110 days.

The days of medication for young survivors diagnosed with colon cancer increased from 14,558 days in 2013 to 25,765 days in 2017; days per person increased from 76 days to 96 days. The days of medication for older survivors increased from 257,610 days in 2013 to 401,315 days in 2017; days per person increased from 91 days to 103 days.

The days of medication for young survivors diagnosed with gastric cancer increased from 20,569 days in 2013 to 31,516 days in 2017; days per person increased from 72 days to 83 days. The days of medication for older survivors increased from 154,979 days in 2013 to 238,099 days in 2017; days per person increased from 81 days to 90 days.

## 4. Discussion

This study analyzed convalescent hospital use among female cancer survivors from 2013 to 2017 by comparing medical expenses, number of visit days, and medication data from the Health Insurance Review and Assessment Service. We found that the number of young and older female cancer survivors who have been treated in convalescent hospitals increased per year, together with increasing medical expenses. A previous study has shown that the annual average rate of visits to acute and convalescent hospitals increased by 3.9% and 6.4% between 2013 and 2017, respectively, in patients treated for gastric cancer [20]. Taken together, these data show that the use of convalescent hospitals for cancer patients is increasing; therefore, quality management, sufficient medical resources, and an efficient medical delivery system are required to provide adequate medical care. 

Cancer treatment in acute medical institutions is typically inpatient and outpatient treatment for surgery, chemotherapy, radiation therapy, and targeted therapy. However, cancer survivors experience side effects, damage to their immune system [4], decreases in body function [3], and endocrine complications [5] during the long-term treatment period after diagnosis. In addition, cancer survivors report insufficient interest in and support for counseling. Self-management and self-care, according to treatment methods, are difficult, and it is difficult to receive family care; therefore, they use convalescent hospitals [6,12,21,22].

Convalescent hospitals provide long-term medical care for elderly and chronically ill patients; however, the number of convalescent hospitals for cancer patients has increased, which has led to an increase in the use of convalescent hospitals during or after cancer diagnosis and treatment in advanced medical institutions [15]. 

Most cancer survivors admitted to convalescent hospitals are women (76.5%), and they have lower quality of life than men [23]. Physical symptoms, such as loss of appetite, pain, vomiting, and fatigue, and psychological symptoms, such as self-efficacy, anxiety, and depression, are significantly worse in female cancer patients than men [24]. This has led to an increase in convalescent hospital use because women carry an increased burden of responsibility for family and work life, such as childrearing, childbirth, and housework [25]. To date, most studies have been conducted on cancer survivors in acute medical institution settings; however, it is difficult to provide tailored education to the individual needs of cancer survivors in this environment due to limited time, space, and human resources. Considering these points, it is necessary to develop an integrated nursing intervention strategy through the expansion of community management projects, including convalescent hospitals, so that difficult symptom management can be successfully adapted to daily life during and after treatment [26]. In addition, the provision of optimized help via differentiated symptom relief and management strategies to older female cancer patients is required because they are more vulnerable in terms of symptoms than men.

In this study, breast and gastric cancer showed higher rates of increase in older cancer survivors than in younger cancer survivors. Emotional support, normal weight maintenance, eating a healthy diet, and physical activity are important factors that influence the happiness and prognosis of cancer survivors as age increases [9,11]. However, it is difficult to provide appropriate care at home due to decreased physical strength and the independence of children as they age. This leads to admission to a convalescent hospital as a solution to food, clothing, and shelter [26]. Therefore, an integrated approach that includes physical, psychological, and social aspects is crucial for the management of physical and psychological symptoms.

Unlike Western countries, there is a high incidence of breast cancer in younger patients in Korea [1]. As a result, young cancer survivors experience physical, mental, and social discomfort over a longer period [2]. In particular, breast cancer survivors have higher anxiety and depression at a younger age [27]. Furthermore, women under 50 years of age who survive colon cancer have higher anxiety and depression [28]. In patients who are diagnosed with colon cancer, proper exercise and a balanced diet are factors that influence recurrence and mortality rates [29]; however, women report less lifestyle improvements than men [30]. It is hypothesized that females experience greater difficulties because they still play the role of caregivers themselves [26]. Therefore, female cancer survivors require a differentiated approach from men in order to adapt and improve their daily life after cancer diagnosis.

The Health Insurance Review and Assessment Service recommends that cancer treatment costs and length of stay of each hospital are disclosed and only high-risk and acute treatments are admitted to advanced medical institutions to reduce medical expenses, shorten hospitalization period, decrease the number of waiting patients, and increase ward turnover [12]. Some cancer patients receive cancer treatment at an acute hospital, followed by admission to a convalescent hospital for rehabilitation, including complementary and alternative therapies, and receive treatment periodically at an acute hospital [21,23]. 

There is a significant gap in cancer mortality according to socioeconomic factors [17], and 51% of cancer survivors use complementary and alternative therapies, especially young women and those with high education levels [31]. In convalescent hospitals, immunotherapy, thermal therapy, meditation, music therapy, art therapy, healthy diet, and nutrition therapy alleviate pain in cancer patients and have a positive effect on the quality of life by improving comfort [15,23,32,33]. Future studies should focus on identifying the determinants for female cancer survivors to visit convalescent hospitals, medical provider factors, and institutional factors. This will establish a model for the friendly and efficient use of convalescent hospitals [34]. In addition, due to the increased number of convalescent hospitals, there is a large difference in quality between institution increases in medical expenses [35]. Therefore, it is necessary to improve universal policies and systems, along with quality management certification programs, so that convalescent hospital use does not become representative of cancer socioeconomic inequality.

The strength of this study is based on national data, which distinguishes between young and older female survivors of breast, colon, and stomach cancer in Korea over 5 years, which compared medical use to suggest policy and system improvement. In addition, the results of this study have confirmed the need to develop standard guidelines for inpatient management in convalescent hospitals and the health management of female cancer survivors by cancer type. 

There are some limitations of this study. First, we only included the medical records of patients who used convalescent hospitals for breast, colon, or stomach cancer. These were chosen as they represent the cancers with the highest incidence in females in Korea, based on claimed health insurance data. However, non-covered treatment and expenses, oriental medicine treatments, and complementary and alternative therapies performed in convalescent hospitals were not included. Furthermore, it was not possible to confirm convalescent hospital use in all female cancer patients.

## 5. Conclusions

This study analyzed convalescent hospital use among female cancer survivors for 5 years by comparing medical expenses, number of visit days, and medication data based on national health data. The annual rate of convalescent hospitalization was higher in older women (8.29~16.39%) than in younger women (4.01~7.46%). The total yearly medical expenses of cancer survivors in convalescent hospitals increased in both age groups and all cancer types, and the range of increase rate was from 7.7% in young breast cancer survivors to 32.2% in young colon cancer survivors. Visit days and days of medication increased noticeably in young colon cancer survivors. The findings of this study are that the number of young and older female cancer survivors who have been treated in convalescent hospitals has increased per year, together with increasing medical expenses. In addition, the annual rate of convalescent hospitalization was higher in older women than in younger women. This study has confirmed the importance of developing standard guidelines for inpatient management in convalescent hospitals and the health management of women cancer survivors by cancer type.

## Figures and Tables

**Table 1:** Number of cancer survivors in convalescent hospitals by age group.

	Type	Breast Cancer		Colon Cancer		Stomach Cancer	
Year		Young	Older	Young	Older	Young	Older
2013		1942	2736	192	2821	285	1919
2014		2196	3440	246	3091	329	2149
2015		2249	3941	231	3381	378	2347
2016		2282	4379	244	3604	365	2540
2017		2557	5021	267	3880	380	2648
Average annual rate of increase (%)		4.01	16.39	8.59	8.29	7.46	11.49

**Table 2:** Total medical expenses for female cancer survivors in convalescent hospitals by age group between 2013 and 2017 (1000 KRW ≒ 1 USD).

Type Year	Breast Cancer				Colon Cancer				Stomach Cancer			
	Young		Older		Young		Older		Young		Older	
	Medical Expenses (per Person)											
2013	9,656,575	(4972)	15,165,249	(5542)	751,141	(3912)	17,002,601	(6027)	1,081,427	(3794)	10,050,089	(5237)
2014	11,302,618	(5146)	20,072,768	(5835)	1,021,191	(4151)	20,157,290	(6521)	1,242,347	(3776)	11,255,069	(5237)
2015	11,631,094	(5171)	23,480,112	(5957)	1,129,873	(4891)	22,495,030	(6653)	1,571,036	(4156)	13,309,720	(5670)
2016	12,358,923	(5415)	28,347,683	(6473)	1,161,927	(4761)	25,510,412	(7078)	1,680,181	(4603)	14,494,881	(5706)
2017	13,696,931	(5356)	31,253,272	(6224)	1,380,699	(5171)	28,059,943	(7231)	1,655,048	(4355)	16,172,853	(6107)

**Table 3:** Visit days for women cancer survivors in convalescent hospitals by age group between 2013 and 2017.

Type Year	Breast Cancer				Colon Cancer				Stomach Cancer			
	Young		Older		Young		Older		Young		Older	
	Days (per Person)											
2013	191,610	(99)	273,948	(100)	14,277	(74)	256,966	(91)	20,334	(71)	152,901	(80)
2014	214,486	(98)	357,006	(104)	19,263	(78)	301,593	(98)	23,329	(71)	169,820	(79)
2015	214,555	(95)	408,500	(104)	21,008	(91)	329,689	(98)	28,347	(75)	198,608	(85)
2016	227,064	(99)	486,650	(111)	21,786	(89)	366,612	(102)	30,484	(84)	213,833	(84)
2017	249,870	(98)	538,563	(107)	25,168	(94)	393,834	(102)	30,317	(80)	232,608	(87)

**Table 4:** Days of medication for women cancer survivors in convalescent hospitals by age group between 2013 and 2017.

Type Year	Breast Cancer				Colon Cancer				Stomach Cancer			
	Young		Older		Young		Older		Young		Older	
	Days (per Person)											
2013	194,352	(100)	277,576	(101)	14,558	(76)	257,610	(91)	20,569	(72)	154,979	(81)
2014	218,206	(99)	362,302	(105)	19,597	(80)	305,571	(99)	23,961	(73)	172,600	(80)
2015	220,642	(98)	419,945	(107)	21,838	(95)	338,063	(100)	29,496	(78)	204,287	(87)
2016	236,567	(104)	504,983	(115)	23,045	(94)	374,574	(104)	31,330	(86)	218,780	(86)
2017	259,978	(102)	554,693	(110)	25,765	(96)	401,315	(103)	31,516	(83)	238,099	(90)
